# Non-traumatic Intramural Duodenal Hematoma With Acute Pancreatitis in a Nonagenarian: A Case Report

**DOI:** 10.7759/cureus.106277

**Published:** 2026-04-01

**Authors:** Toshifumi Shinbo, Yasumasa Nakasha, Kaname Ishiguro

**Affiliations:** 1 Department of Surgery, Noto General Hospital, Nanao, JPN

**Keywords:** acute pancreatitis, anticoagulant therapy, duodenal obstruction, elderly patient, gastrojejunostomy, intramural duodenal hematoma, non-traumatic hematoma

## Abstract

Intramural duodenal hematoma (IDH) is a relatively rare condition typically managed conservatively. Although IDH frequently causes duodenal obstruction, the development of acute pancreatitis (AP) due to ampulla of Vater compression is uncommon. We report the case of non-traumatic IDH in a 90-year-old man receiving anticoagulant therapy, complicated by AP and persistent duodenal obstruction. Imaging studies revealed an intramural hematoma in the descending portion of the duodenum, without evidence of active bleeding. Despite five weeks of conservative management, the obstruction did not improve, and progressive malnutrition was a concern. Therefore, a gastrojejunostomy was performed without hematoma evacuation. The postoperative course was uneventful, and subsequent imaging confirmed spontaneous resolution of the hematoma. This case highlights that IDH can be associated with AP and that gastrojejunostomy without hematoma removal may be a viable surgical option in selected cases with persistent obstruction.

## Introduction

Intramural duodenal hematoma (IDH) can have various causes [1-5]. Although the exact incidence remains unclear, this condition is rare, and anticoagulant therapy is a possible cause. IDH often results in duodenal obstruction; however, it may also be complicated by acute pancreatitis (AP), potentially triggered by the compression of the ampulla of Vater by the hematoma. Several mechanisms have been proposed to explain this association, and these pathophysiological processes were classified by Shiozawa et al. [6].

Conservative management is generally considered the mainstay of treatment for IDH, and in most cases, the hematoma resolves spontaneously within 1-3 weeks [7]. Nevertheless, when obstruction persists, invasive interventions should be considered. In older patients, careful assessment of the balance between surgical invasiveness and risks associated with prolonged conservative management is essential.

We report the case of non-traumatic IDH in a 90-year-old man receiving anticoagulant therapy, complicated by duodenal obstruction and AP, which was presumably caused by papillary compression. In this case, gastrojejunostomy was performed without hematoma evacuation, and spontaneous resolution of the hematoma was subsequently confirmed. This case illustrates the complexity of determining the optimal surgical strategy and the timing of intervention in older patients.

## Case presentation

A 90-year-old male patient presented to our outpatient department with abdominal pain and vomiting. His medical history included atrial fibrillation, for which he was prescribed apixaban. The patient exhibited a high degree of autonomy in daily activities and had no history of trauma. On presentation, his vital signs were as follows: body temperature, 35.6°C; blood pressure, 75/52 mmHg; heart rate, 79 beats/min; and oxygen saturation, 92% on room air, indicating shock. Physical examination revealed a tender, palpable mass in the right upper abdomen. The laboratory findings were as follows: white blood cell count, 18,500/μL; hemoglobin level, 11.0 g/dL; platelet count, 370×10³/μL; serum amylase level, 1,066 U/L; C-reactive protein level, 4.81 mg/dL; and prothrombin time-international normalized ratio, 1.26. The liver enzymes and bilirubin levels were within normal limits (Table 1).

**Table 1:** Serial laboratory data during hospitalization AST: aspartate aminotransferase; ALT: alanine aminotransferase; ALP: alkaline phosphatase; GGT: gamma-glutamyl transferase; BUN: blood urea nitrogen; eGFR: estimated glomerular filtration rate; CRP: C-reactive protein; INR: international normalized ratio

Laboratory parameter	Day 1	Day 8	Day 18	Day 29	Preoperative (day 35)	Postoperative (day 38)	Reference range
Hemoglobin (g/dL)	11.0	6.8	8.0	8.8	9.0	7.2	13.7-16.8
Platelet count (×10^3^/μL)	370	317	464	376	375	276	158-348
AST (U/L)	13	20	20	21	20	19	13-30
ALT (U/L)	13	16	16	13	12	10	10-42
ALP (U/L)	95	89	281	119	125	-	38-113
GGT (U/L)	10	34	81	31	24	-	13-64
Total bilirubin (mg/dL)	1.4	1.6	1.8	1.1	1.9	1.5	0.4-1.5
Amylase (U/L)	1,066	43	51	37	53	40	44-132
Albumin (mg/dL)	3.6	2.2	-	2.6	3.0	-	4.1-5.1
BUN (mg/dL)	33	7	9	20	41	47	8-20
Creatinine (mg/dL)	1.45	0.83	0.81	0.81	0.87	1.19	0.65-1.07
eGFR (mL/min/1.73 m^2^)	35.5	65.4	67.2	67.2	62.1	44.1	≥60
CRP (mg/dL)	4.81	10.80	5.65	0.76	0.47	4.02	0-0.14
INR	1.26	-	1.43	1.35	1.33	-	1.0-1.1

**Figure 1:**
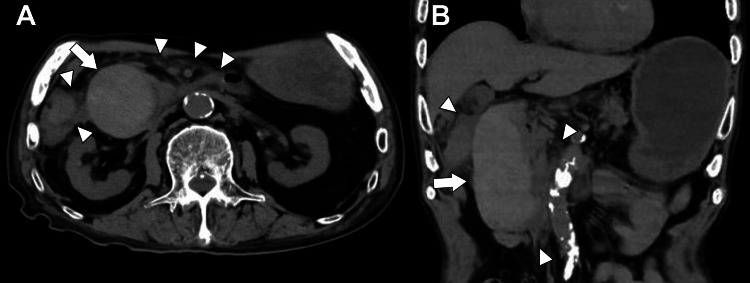
Abdominal computed tomography on day 1 (A) Axial view and (B) coronal view show an intramural hematoma in the descending portion of the duodenum (arrows), with periduodenal and peripancreatic fat stranding and fluid collection (arrowheads).

Considering the renal dysfunction, contrast-enhanced imaging was avoided, and non-contrast computed tomography was performed. This revealed an intramural hematoma in the descending portion of the duodenum, along with increased fat stranding around the duodenum and pancreas (Figure 1A, 1B).

**Figure 2:**
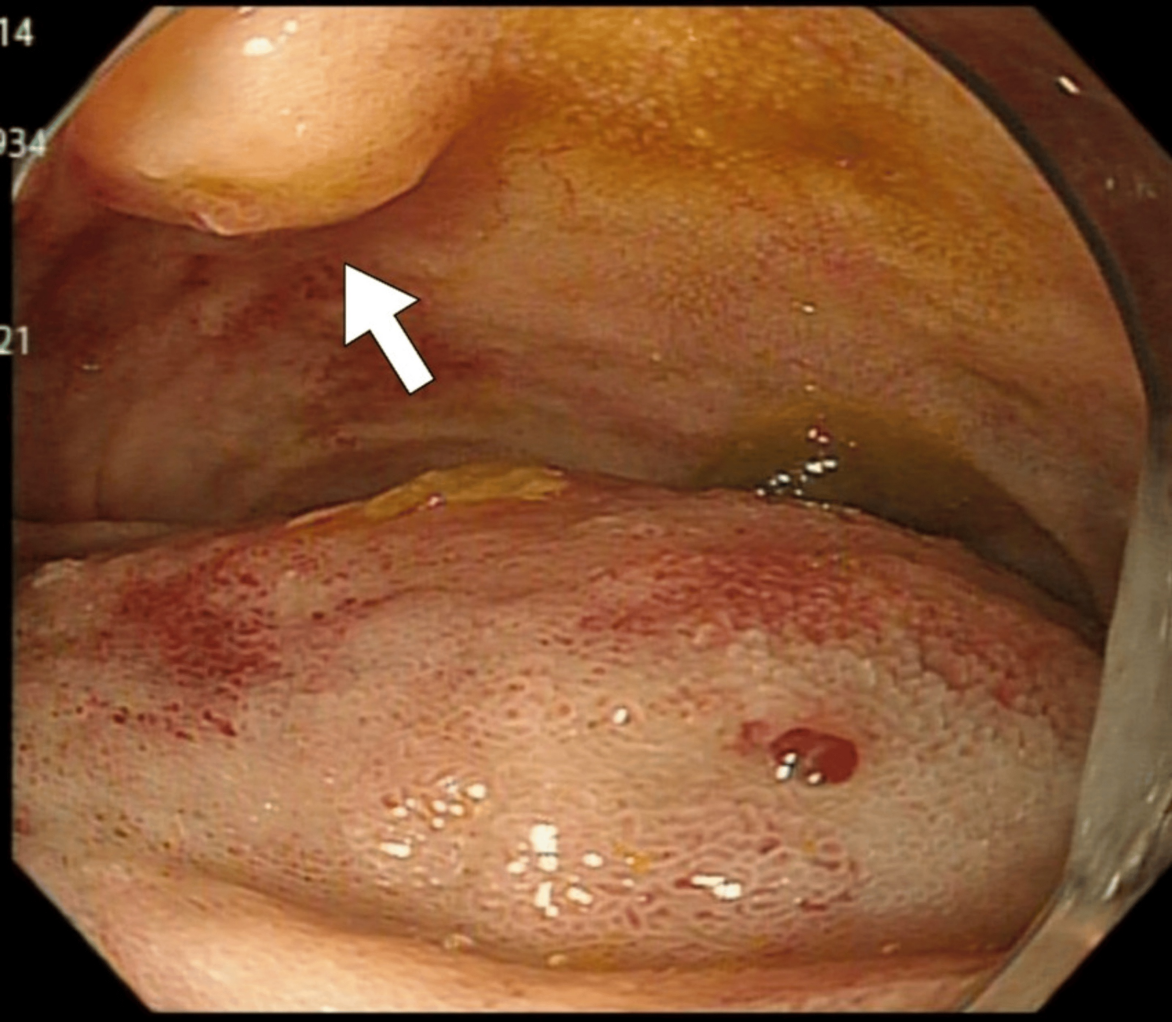
Upper gastrointestinal endoscopy on day 8 An intramural hematoma in the descending portion of the duodenum, located opposite the ampulla of Vater (arrow).

No evidence of active bleeding was observed. The patient was diagnosed with duodenal obstruction and AP, the latter being a secondary complication of IDH. The patient was admitted to our hospital for conservative management. Because of circulatory instability, prompt fluid resuscitation was initiated, resulting in the stabilization of his hemodynamic status. Apixaban was discontinued, and conservative treatment, including fasting and parenteral nutrition, was initiated. The pancreatitis gradually improved. Subsequent upper gastrointestinal endoscopy on day 8 revealed an intramural hematoma in the descending duodenum, located opposite the ampulla of Vater (Figure 2).

**Figure 3:**
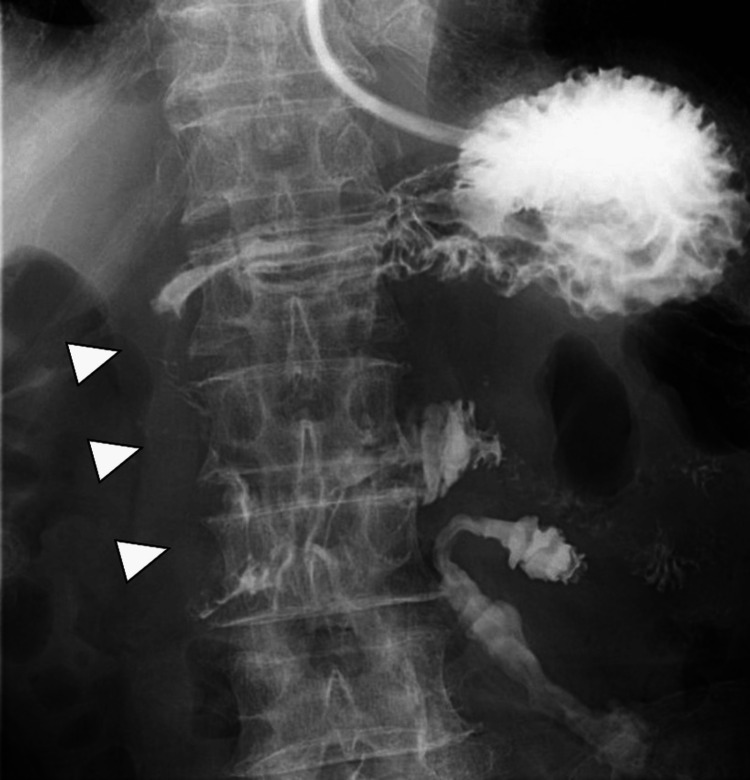
Contrast study through the nasogastric tube on day 29 Persistent stenosis of the descending portion of the duodenum (arrowheads), with minimal passage of the contrast medium.

The endoscope barely passed through the stenotic segment. Oral intake was initiated on day 9; however, persistent vomiting made continued intake difficult, even with water. A nasogastric tube was therefore inserted for gastric decompression. On day 18, the patient developed a fever. Contrast-enhanced computed tomography led to a diagnosis of mild cholangitis; however, no aneurysm was identified as the source of the hematoma. The cholangitis improved promptly with antibiotic therapy. Spontaneous resolution of the hematoma was anticipated; however, no discernible improvement was evident after four weeks. Fluoroscopic examination on day 29 revealed persistent stenosis with minimal passage of the contrast medium (Figure 3).

**Figure 4:**
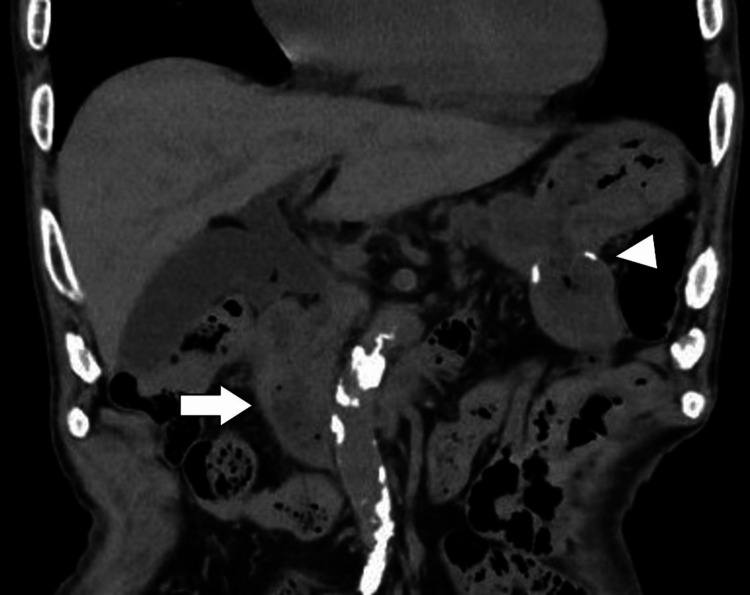
Abdominal computed tomography on day 77 Near-complete resolution of the intramural duodenal hematoma. The duodenum (arrow) and the gastrojejunostomy site (arrowhead) are indicated.

Following a multidisciplinary discussion, an endoscopic intervention was considered; nevertheless, this approach was not pursued because of the perceived complexity of managing the complications that might arise during the procedure. Considering the failure of prolonged conservative therapy, surgical intervention was performed on day 37. Given the patient's advanced age and reduced physical reserve, hematoma evacuation was considered a high-risk procedure for extensive duodenal wall injury. Consequently, gastrojejunostomy was performed without concomitant hematoma removal. The postoperative course was characterized by no significant complications, and the patient was discharged on postoperative day 28 (hospital day 65). On day 77 (12 days after discharge), the patient was followed up in the outpatient department. Mild physical deconditioning was noted; however, no notable complications were observed, and oral intake remained adequate. Computed tomography performed on the same day revealed near-complete resolution of the hematoma (Figure 4).

## Discussion

AP development secondary to IDH is uncommon. Advanced age is a risk factor in the severity assessment of AP [8], necessitating the meticulous systemic management of older patients. In cases of persistent obstruction, gastrojejunostomy without hematoma evacuation can be considered a less invasive surgical option.

Duodenal hematomas most commonly result from trauma; however, they have reportedly occurred following endoscopic procedures [1]. Non-traumatic cases have been associated with anticoagulant therapy [2], pancreatitis [3], pancreatic cancer [4], and ruptured aneurysm [5]. In this particular case, there was no history of trauma, and no bleeding source, such as an aneurysm, was identified through imaging. Anticoagulant therapy was the most likely contributing factor. The most common location of an IDH is the second and third portions of the duodenum, likely because these segments are relatively fixed in the retroperitoneum and have a rich submucosal blood supply via the superior and inferior pancreaticoduodenal arteries [9]. In patients receiving anticoagulation therapy, spontaneous hemorrhage into the submucosal layer may occur without a discrete vascular rupture, resulting in intramural hematoma formation [6]. Although hematoma rupture is possible, it typically extends into the peritoneal or retroperitoneal cavity; rupture into the duodenal lumen is rare [10]. Shiozawa et al. classified duodenal hematomas associated with pancreatitis into three types: (a) obstruction of the duodenal papilla by the hematoma, (b) hematoma formation due to vascular disruption by pancreatic enzymes released during AP, and (c) hematoma formation due to vascular disruption by pancreatic enzymes released during chronic pancreatitis or its acute exacerbation [6]. However, distinguishing between these mechanisms is challenging [10]. In this case, a submucosal hematoma located opposite the ampulla likely compressed the common pancreaticobiliary channel, resulting in obstructive pancreatitis, corresponding to type (a) in the Shiozawa classification.

Conservative management is recommended as the mainstay treatment in the absence of perforation or bleeding. Spontaneous resolution generally occurs within 1-3 weeks [7], although longer durations have been reported [11,12]. In cases that are refractory to conservative treatment or accompanied by complications, invasive interventions should be considered. When active bleeding or an underlying aneurysm is identified, interventional radiology is indicated for hemostasis [13]. Once hemostasis has been achieved, percutaneous or endoscopic drainage [14-16], surgical hematoma evacuation [17], or gastrojejunostomy may be considered. In this particular instance, conservative therapy was pursued for approximately five weeks, with the objective of circumventing the necessity for invasive intervention in an older patient with concomitant AP. However, the obstruction persisted, with concerns regarding malnutrition and complications related to prolonged central venous catheterization. The use of endoscopic intervention was considered; however, it was not pursued because of the challenges associated with the adequate management of potential complications arising from the procedure. Consequently, surgical intervention was deemed the optimal course of action. The obstruction was promptly relieved by gastrojejunostomy. However, subsequent confirmation of spontaneous resolution of the hematoma suggests that continued conservative management alone might have eventually led to improvement. This underscores the inherent complexity of ascertaining the optimal timing of intervention in such cases. In cases such as the present one, where duodenal obstruction persists, deterioration of nutritional status due to prolonged fasting, an increased risk of infection associated with long-term central venous catheter placement [18], and an increased risk of thrombosis should be considered. When considering intervention, it is important to comprehensively evaluate factors such as (1) persistent obstruction, (2) deterioration of nutritional status, and (3) catheter-related complications. Although approximately three weeks may serve as a general reference point, decisions should be made on an individual basis based on these factors.

## Conclusions

We present a rare case of non-traumatic IDH complicated by AP in an older patient receiving anticoagulant therapy. This case provides three important clinical insights. First, IDH may contribute to the development of AP through the compression of the ampulla of Vater. Second, when obstruction persists, minimally invasive surgical options, such as gastrojejunostomy without hematoma evacuation, may be considered in addition to continued conservative management. Third, the timing of intervention should be determined not solely based on the duration of symptoms, but through a comprehensive assessment of factors such as persistent obstruction, deterioration of nutritional status, and catheter-related complications.
